# Multi-radial LBP Features as a Tool for Rapid Glomerular Detection and Assessment in Whole Slide Histopathology Images

**DOI:** 10.1038/s41598-018-20453-7

**Published:** 2018-02-01

**Authors:** Olivier Simon, Rabi Yacoub, Sanjay Jain, John E. Tomaszewski, Pinaki Sarder

**Affiliations:** 1 Department of Pathology and Anatomical Sciences, University at Buffalo, Buffalo, USA; 2 Division of Nephrology, Department of Medicine, University at Buffalo, Buffalo, USA; 3 Division of Nephrology, Department of Medicine, Washington University School of Medicine, St. Louis, USA

## Abstract

We demonstrate a simple and effective automated method for the localization of glomeruli in large (~1 gigapixel) histopathological whole-slide images (WSIs) of thin renal tissue sections and biopsies, using an adaptation of the well-known local binary patterns (LBP) image feature vector to train a support vector machine (SVM) model. Our method offers high precision (>90%) and reasonable recall (>70%) for glomeruli from WSIs, is readily adaptable to glomeruli from multiple species, including mouse, rat, and human, and is robust to diverse slide staining methods. Using 5 Intel(R) Core(TM) i7-4790 CPUs with 40 GB RAM, our method typically requires ~15 sec for training and ~2 min to extract glomeruli reproducibly from a WSI. Deploying a deep convolutional neural network trained for glomerular recognition in tandem with the SVM suffices to reduce false positives to below 3%. We also apply our LBP-based descriptor to successfully detect pathologic changes in a mouse model of diabetic nephropathy. We envision potential clinical and laboratory applications for this approach in the study and diagnosis of glomerular disease, and as a means of greatly accelerating the construction of feature sets to fuel deep learning studies into tissue structure and pathology.

## Introduction

Virtual histopathology, in which histology slides are digitally scanned at high resolution and stored as whole-slide images (WSIs), has rapidly assumed a prominent role in pathology research. It allows for easy sharing and storage of tissue structure information without many of the drawbacks of working with the original glass slides, such as their fragility, bulkiness, and observer variability due to illumination differences^1–3^. At the same time, the rapid evolution of machine learning methods has made image-based computer-aided diagnosis increasingly practicable, particularly for the analysis of light microscopy WSIs, dermatoscopy images, and radiological data^4–6^. Such methods include support vector machines (SVMs), boosted decision trees, artificial neural networks, and non-negative matrix factorization methods^7^, all of which have the potential for condensing very subtle and high-dimensional image features into relatively simple and decisive pathologic classifications^8^.

The glomerulus, a histologic structure found in the cortex of the kidney, forms the initial interface for filtration of metabolic wastes from the bloodstream. Glomeruli are roughly spherical and contain tightly packed loops of capillaries juxtaposed against a convoluted collagenous sheath (the basement membrane) which is supported by podocytes and functions as the primary filter. An intervening supportive structure, the mesangium, assists in regulating blood flow and readily takes up common histological stains such as Periodic Acid Schiff (PAS)^9,10^.

Given its crucial role and structural intricacy, diseases of the glomerulus are widespread and can have devastating impact. Although many varieties are known, such as, IgA nephropathy and lupus nephritis, glomerular disease is a particularly common as a co-morbidity of diabetes, mainly in the form of diabetic nephropathy (DN). In DN, thickening of the basement membrane, expansion of the mesangium and overall loss of the podocytes and filtration boundary leads first to microproteinuria and then, in approximately 4–17% of type II diabetic cases, renal failure and end-stage renal disease (ESRD)^10,11^. Importantly, owing to the dramatic increase in diseases of glucose metabolism such as metabolic syndrome and type 2 diabetes (anticipated to afflict 37% of the US population), DN is projected to greatly increase in prevalence^12^.

Currently, assessment of DN depends on manual annotation by pathologists, which is time-consuming and represents a significant bottleneck and expense in the treatment pipeline. Furthermore, despite general agreement on the hallmarks of glomerular damage in DN, pathologists often disagree on scoring and diagnosis of the condition^10^. Finally, the progression of DN is subject to dramatic differences in progression rates, with some cases rapidly progressing to ESRD while others spontaneously regress^9,11^. These factors combine to create unusual prognostic difficulties which, along with DN’s large and growing prevalence, underscores the desirability of automated tools to facilitate the assessment of DN from histopathology data. Yet, though machine learning techniques have been applied to other diabetic complications, such as diabetic retinopathy^13^ and diabetic neuropathy^14^, and semi-automated segmentation of glomeruli^15,16^, fully supervised learning techniques for efficient glomerular localization in WSIs remain an open challenge.

To this end, a wide assortment of textural classifiers have been mustered in the search for optimal supervised classification of image features, including histogram of oriented gradients (HOG), local binary patterns (LBP), concurrence matrices, grayscale histograms, chromaticity moments and Gabor wavelets^17,18^. Approaches to glomerular detection have also made use of genetic algorithms^16,19,20^ and large Internet databases such as Cytomine^21^, yet these methods are too computationally intensive or offer limited performance. Afield of machine learning, staining with antibodies to the intermediate filament nestin can highlight glomeruli^22^, while in MRI, paramagnetic nanoparticles can outline the glomerular basement membrane^23^; however, such label-based methods are laborious and suffer from high variability and noise^24^.

**Figure 1:**
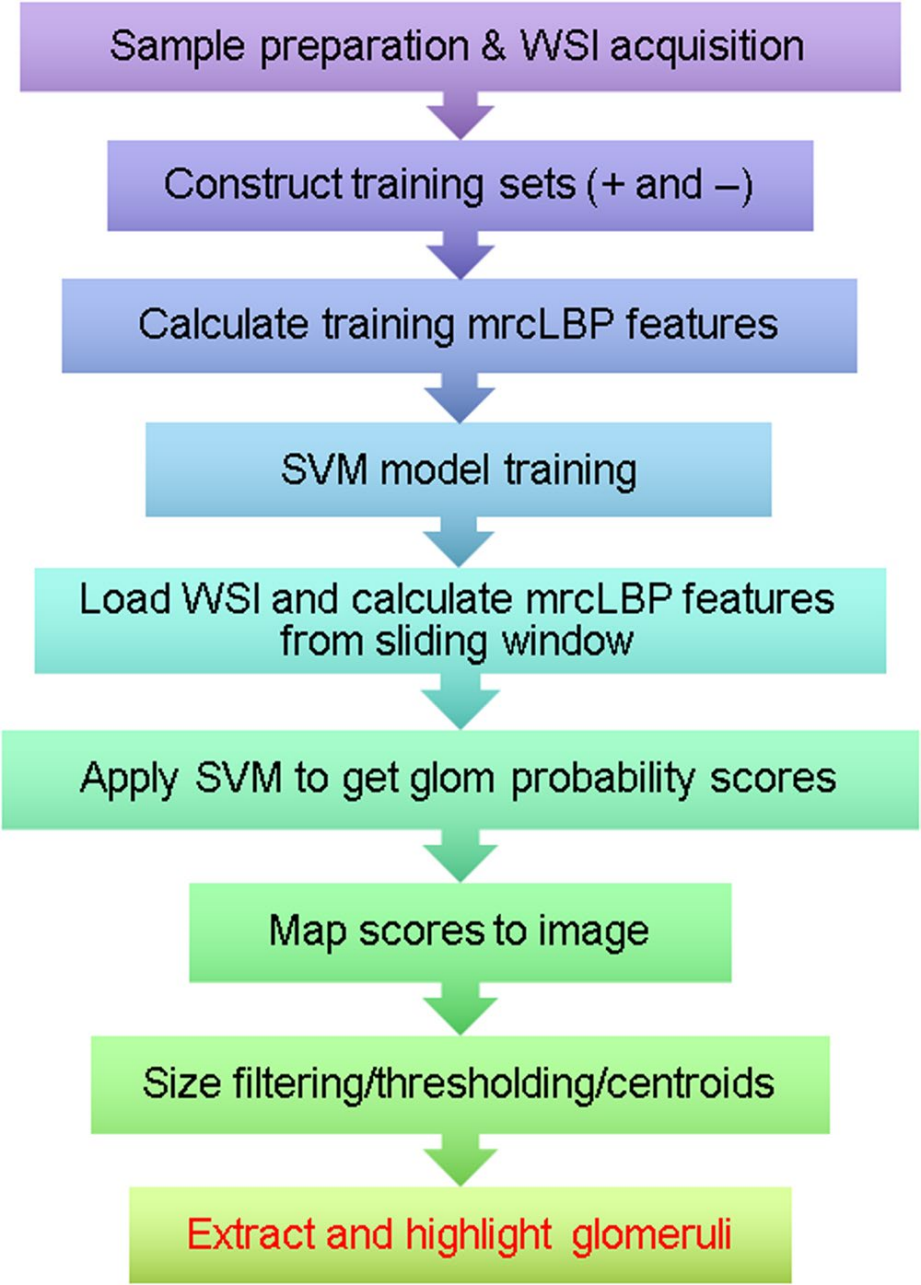
Schematic of pipeline involved in glomerular detection. Samples are first prepared, training sets are gathered by hand and mrcLBP feature vectors are calculated from them. Next, an SVM classifier is trained using these sets and deployed via a sliding window on the WSI of interest to give a map of glomerulus detection scores over the slide. This map is thresholded and filtered to remove objects too large or small to be glomeruli, centroids are calculated for the remaining dark regions, and the glomeruli are highlighted or automatically cropped.

Here, we employ the LBP texture descriptor, first described in 1994^25^, in a supervised-learning pipeline to achieve glomerular detection (Fig. 1) and also to distinguish between disease and control glomeruli in a mouse model of DN. In brief, LBP works by defining a circular set of “neighbor” pixels at a fixed radius from each image pixel, then thresholding each neighbor with respect to the central pixel; those with intensity greater than or equal to the center pixel are thus assigned “1” values, while those with lower intensity are assigned “0”s. The resulting binary string is a simple and rapidly calculable local descriptor, which can then be binned and normalized to profile the frequency of each pattern type in the image. The output is usually further simplified by only distinguishing among “uniform” LBPs–those patterns in which there are at most two transitions between “0” and “1”–as these typically contain the vast majority of textural information^25^. Because of its localized thresholding, LBP is notably robust to variations in image illumination^26^. Moreover, LBPs can readily be adapted for “multi-resolution analysis” or “multidimensional LBP” by performing LBP at multiple radii or resolutions^25,27^.

**Figure 2:**
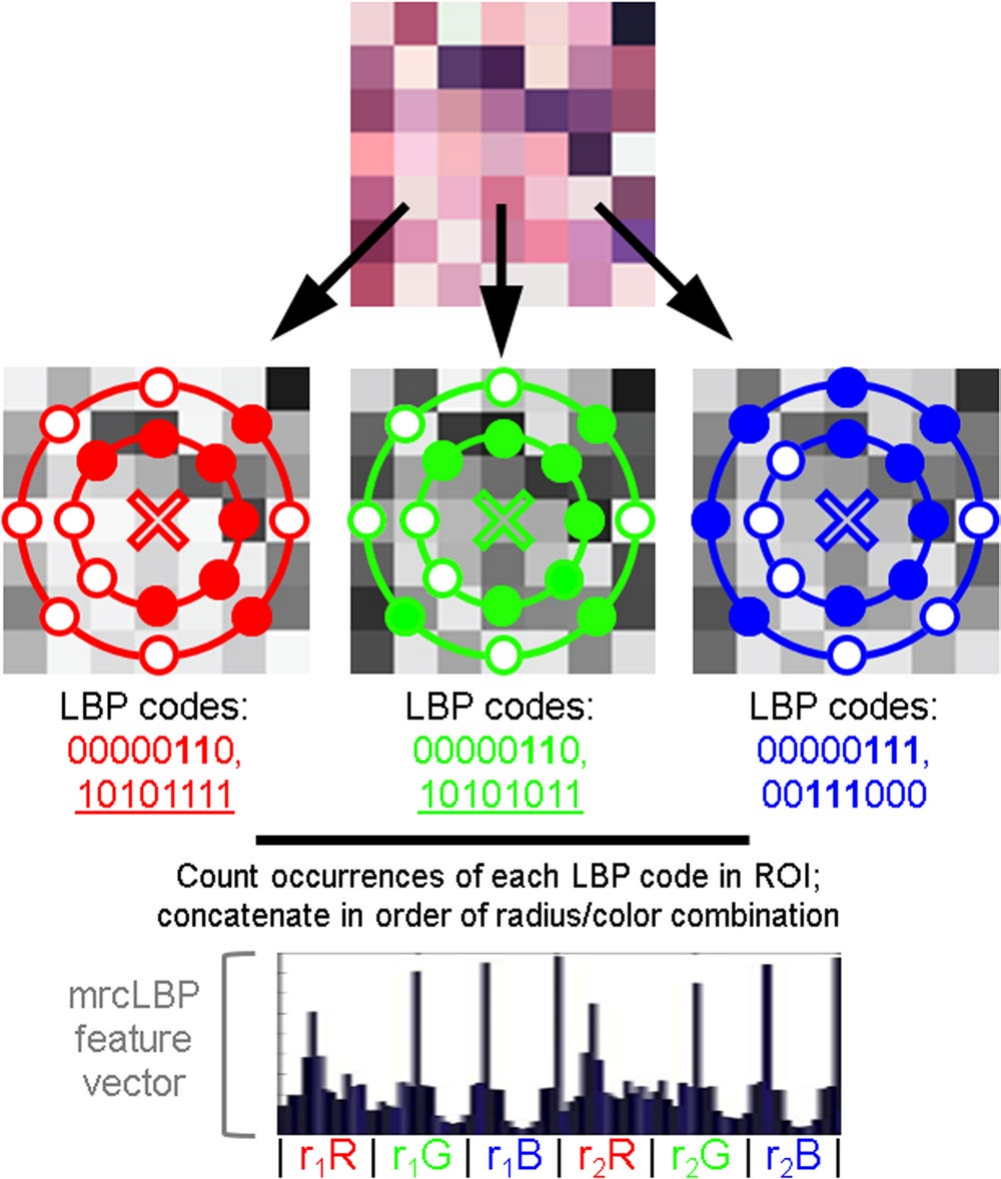
Overview of the procedure for mrcLBP feature vector construction. The LBP feature is derived from the intensity of a circular neighborhood of eight pixels thresholded by a central pixel (marked “X”). Pixels less intense than the central pixel are assigned a value of 0 (filled), and 1 otherwise (white) to generate a distinctive binary code. The occurrences of these codes can then be counted over the training image region of interest. In mrcLBP, this process is repeated with multiple radii for each of the RGB color channels, and the results from each of these combinations are concatenated to create a new feature vector.

It has been observed in many cases that such multidimensional methods can markedly improve the texture-recognition capabilities of LBPs^27^; therefore, in this work, we employed a similar approach, referred to here as multi-radial color LBP (mrcLBP). Briefly, after gathering a training set of glomerular images, we calculate LBP descriptors over a region of each training image corresponding to the center of the glomerulus, repeating the process for four different radii per center pixel, as well as for each RGB color channel. We then concatenate the LBP profiles from the resulting 12 radius-color combinations to create a feature vector for each training image (Fig. 2). Next, the resulting series of mrcLBP feature vectors is used to train an SVM classifier, which can be deployed on other WSIs for glomerular detection. If desired, a trained deep neural network model can be applied, increasing the overall precision further. Finally, by applying mrcLBP to training sets composed of glomeruli gathered from a mouse model of DN, we find evidence that mrcLBPs may be useful not only in automated detection of glomeruli, but in assessing their pathologic condition. Past literature is rich with feature extraction techniques to classify biological patterns in diverse applications, including prostate cancer Gleason grading^28^ and breast cancer grading and nuclei classification^29–31^, to name a few. Moreover, LBP feature vectors have been widely used for classifying tissue types in biological images^32,33^. To our knowledge, however, our work is the first demonstration of mrcLBP feature analysis and SVM classification to localize glomeruli from tubular background in whole slide renal histopathology images of murine, rat, and human tissue images, from diverse histological stains.

## Methods

### Tissue Section Preparation

#### Mouse tissue slices

A standard streptozocin (STZ) mouse model^34^ was used to model diabetes-related kidney damage. C57BL/6 J background mice (7 weeks old) were injected with STZ, producing a mild form of type I diabetes, and after 25 weeks, mild-moderate DN. Control C57BL/6 J mice (7 weeks old) were treated with STZ vehicle. All animal studies were performed in accordance with protocols approved by the Institutional Animal Care and Use Committee at University at Buffalo, and are consistent with federal guidelines and regulations and in accordance with recommendations of the American Veterinary Medical Association guidelines on euthanasia. Kidney tissues were gathered from 10 control and 7 STZ-treated mice. Tissues were fixed with formalin, embedded in paraffin blocks, and cut to 2 μm slice thickness along the sagittal plane using an Olympus CUT 4060 microtome. Slices were then stained with haematoxylin and eosin (H&E).

#### Rat tissue slices

Rat tissue sections (a gift from Dr. Tracey Ignatowski, Pathology and Anatomical Sciences, University at Buffalo) were prepared using the same protocols as for mouse, except that in addition to H&E, Periodic acid-Schiff (PAS), Jones silver, Gömöri’s trichrome, and Congo Red (CR) staining were also carried out. All slices were cut to 5 μm thickness, with the exceptions of the Jones silver and CR slides which were cut to 2- and 8-μm thickness, respectively.

#### Human tissue slices

Biopsy samples from human DN patients with chronic kidney disease stage II and stage III were collected from the Kidney Translational Research Center at Washington University School of Medicine, directed by co-author Dr. Jain. The glomerular structural changes in these biopsies suggest DN-related changes spanning the different DN stages discussed in Tervaert *et al.*^10^. As a control, renal tissue samples of non-diabetic patients with no notable histological abnormalities were used. We used sections with completely normal renal tissues, as verified by Drs. Jain and Tomaszewski. Human data collection followed a protocol approved by the Institutional Review Board at University at Buffalo prior to commencement. All methods were performed in accordance with the relevant federal guidelines and regulations. Participants were required to be over 18 years of age, and with a diagnosis of any renal disease that required renal biopsy. Special populations (vulnerable) such as minors, pregnant women, neonates, prisoners, children, and cognitively impaired patients were not included. All patients provided written informed consent, and basic demographic information was collected. Human tissue slices were cut to 2–5 µm thickness, and stained with PAS.

### Imaging

Imaging was conducted using a whole-slide bright-field microscope (Aperio, Leica, Buffalo Grove, Illinois), with a 40× objective and NA = 0.75; resolution was 0.25 μm∕pixel for all acquired images. For computational tractability, all human WSIs were resized to 50% of original size.

### Training set construction

Two sets of training images were collected from each species, containing centered glomeruli (glom(+)) or no glomeruli (glom(−)). Typically several hundred to a few thousand images of each class were needed for the SVM to achieve good classification performance^8,35,36^. As an excess of negative training images appears to improve precision, all training sets used contained at least twice as many glom(−) images as glom(+). All training images were extracted manually at a size of 576 × 576 pixels, which easily accommodates entire glomeruli.

In total, seven distinct training sets were created, comprising one glom(−) and glom(+) set each from the following: H&E-stained control mice; rat sections in five different staining protocols; and human biopsies and kidney sections. In addition, one glom(+) set was collected from STZ-treated mice. Briefly, the H&E training set for mouse comprises 1059 glom(+) and 1799 glom(−) images gathered from 15 WSIs from healthy mouse kidney. The rat training set consists of images stained with congo, H&E, Jones, PAS, and Gömorri trichrome (three kidney slice WSIs per stain), giving a total of 7099 glom(+) and 15750 glom(−) images from 15 WSIs. Ten WSIs were left for testing, two from each stain type. The human training set consists of 1649 images (515 glom(+), 1144 glom(−)) gathered from a total of 25 WSIs (~2 gigapixels) and one large tissue section (5.25 gigapixels).

### Multi-radial color LBP (mrcLBP)

LBP features were obtained using MATLAB’s *extractLBPFeatures* function, with the ‘upright’ flag was set to ‘false’^37^. This removes the rotation-invariance of the LBP feature, and also reduces its dimensionality from 59 components (one bin for each of the 58 possible uniform LBPs plus 1 bin for all non-uniform LBPs) to 10 components (9 bins for LBPs containing various numbers of 1′s, plus the non-uniform bin). We found no difference in performance using this version with fewer components.

The mrcLBP feature vector for a given image region is constructed by carrying out LBP as above, but on the separate R, G, and B channel, each with four different LBP radii, and concatenating the resulting LBP vectors for each combination of radius and color channel (we used the ordering R1 R3 R9 R27 G1 G3 G9 G27 B1 B3 B9 B27). The resulting 120-dimensional feature vector contains information on textural variations in terms of local intensity differences as a function of color and over a range of scales. Best results were generally achieved using radii of 1, 3, 9, and 27 pixels, though other combinations are also viable for glomerular detection. All experiments involving feature extraction, classifier training, and feature detection were carried out using a pool of 5 Intel(R) Xeon(R) CPU E5-2697 v3 cores @ 2.60 GHz, with 40GB RAM.

### Feature extraction and SVM training

To intercept only the interior of the glomerulus, mrcLBP feature vectors are extracted from the center region (1/6^th^ of the overall width) of the training images. Once both feature and label vectors have been generated from the training set, SVM training is carried out using MATLAB’s *fitcsvm* function, with default parameters^38^.

### Sliding-window extraction of mrcLBP features from test image

For glomerular detection, the WSI image of interest was fed to the trained SVM classifier by way of a standard sliding-window approach, typically with a stride of 64 pixels. Window size is set identical to the size of the training images (576 × 576). In this phase, two main adjustable parameters are involved: the stride, which determines the separation between successive sliding-window positions, and a resizing “calibration” factor that is used to match the resolution (µm/pixel) of the glomeruli in the training images with that of the glomeruli in the WSI being scanned.

### Application of trained SVM to WSI

The trained SVM classifier was deployed on the concatenated mrcLBP feature vectors from each of the sliding window locations, resulting in a score map in which darker pixels (lower values) correspond to a higher classifier-estimated likelihood that the corresponding window region belongs to the glom(+) class.

### Score map processing

To remove large regions with texture similar to the glomeruli (particularly the kidney medulla and certain growths) which can cause false positives, the score map is first thresholded by intensity, then size-filtered using MATLAB’s *bwareaopen* function to remove any dark objects in the map that are too large or small to be glomeruli, and thresholded a second time to better isolate the glomeruli. In most cases, the first intensity threshold was left at 0. The glomerular area range depends on the resolution of the WSI and the stride of the sliding window positions, but was typically 6–50 pixels. The final threshold typically takes a value between 0.15 and 0.35, and once established by trial and error can be applied to many slides without modification. MATLAB’s *regionprops* feature is used to locate the centroids in the remaining regions, which approximate the glomerular centers and allow them to be automatically cropped and saved.

### Deep learning model training for result refinement

For improved-precision multi-stain glomerular detection, a GoogLeNet classification model^39^ was trained in DIGITS with the “Nesterov’s Accelerated Gradient” option, using the full rat training set of 7099 glom(+) images, 15750 glom(−) images. Of these, 20% were randomly assigned to a validation set, and 10% to a test set. Applying this model to the test set, the false positive rate was 0.0019%, and the false negative rate was 0.0042%. Both training and deployment of the GoogLeNet model was carried out using an Nvidia GeForce 1080 GPU.

### Performance testing of glomerular detection

In order to reasonably judge the generality and robustness of the detector, glomerular images being tested are kept distinct from those on which the classifier was trained. After glomeruli were hand-counted to establish ground truth and the SVM false positives were counted, false negative, recall, precision, and F1 scores were calculated directly using standard formulae.

### Characterization of STZ versus control glomeruli

Differences in mrcLBP scores between STZ and non-STZ mice were studied using a method closely similar to that employed in glomerular detection, except that instead of training the SVM on images with or without glomeruli present, the training image sets consisted of glomeruli collected by hand from either control or STZ-treated mice. To assess the robustness of this approach, SVM classifiers were trained using either 10 random cross-validation runs (100 holdout images apiece), and the mean score values of the test images in each case were calculated. The resulting SVM scores were aggregated and displayed as a histogram for both the STZ- and non-STZ holdout images.

## Results

### Glomerular detection in mouse

**Figure 3:**
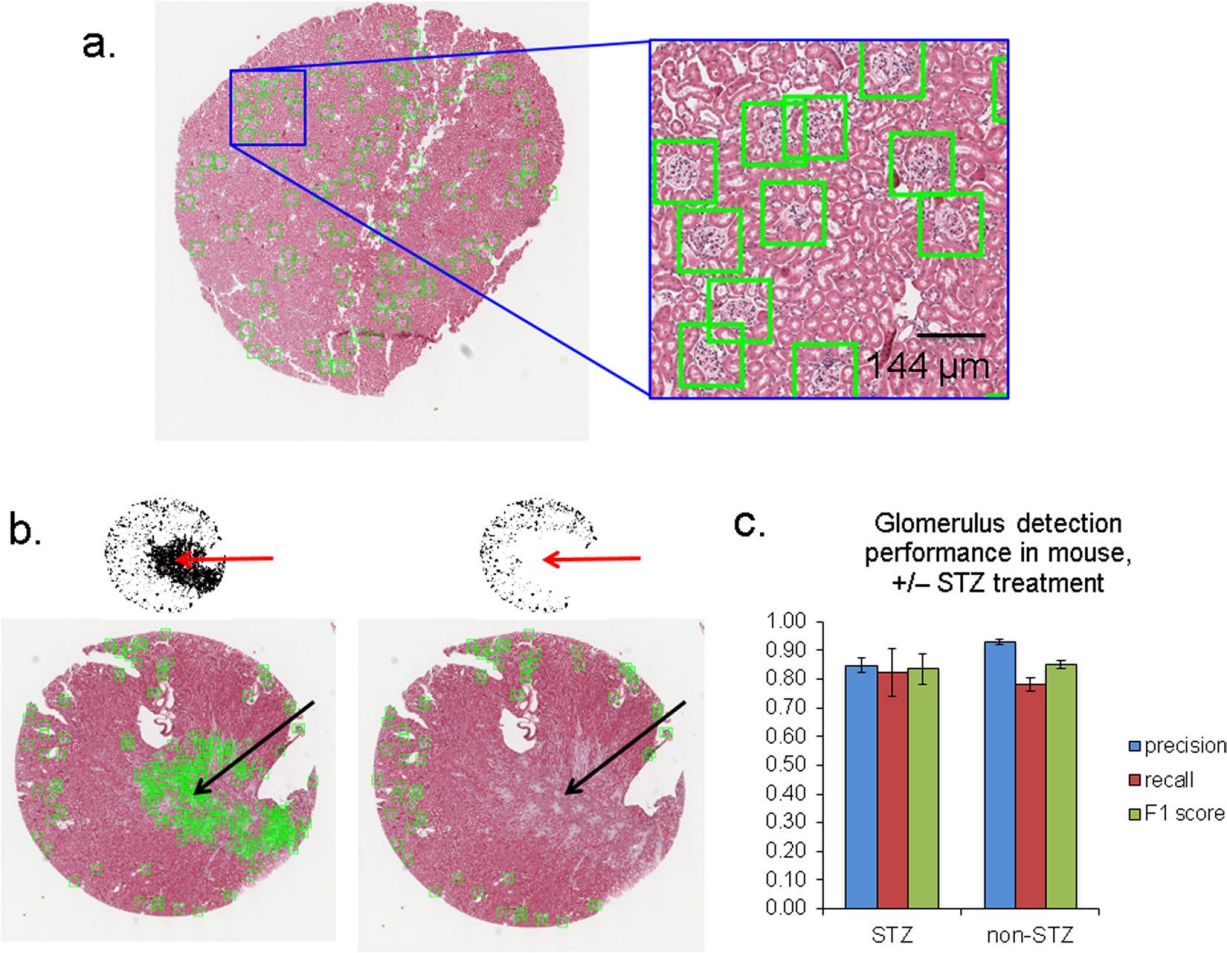
Performance of the mrcLBP glomerular detection pipeline in mouse WSIs. (**a**) Representative mouse WSI with glom locations automatically marked (green squares), H&E stain. Each side of the green squares displayed in this figure (and in all subsequent figures) measures 144 μm. (**b**) Example of removal of large connected areas from the SVM output using area opening. Left, mouse WSI prior to area opening, showing scores map (top) and prospective glomeruli in green boxes (bottom). Right, same slide after successful opening, showing removal of false positives due to a small tumor-like region near the center of the slide and medullar texture at the bottom edge. H&E stain. (**c**) mrcLBP-based glomerulus detection in mouse WSI, with varying degrees of STZ treatment, for a classifier trained on non-STZ glomeruli. Although STZ causes noticeable histological changes in certain glomeruli, the performance of the detection algorithm is only minimally affected, with similar F1-score for all three cases.

Examples of the mrcLBP feature pipeline applied to mouse WSIs are shown in Fig. 3a–c. Although there was some variability in the performance of the method between slides, all showed precision for glomerulus detection in the range of 0.83–0.98, with an average of just over 90% (in terms of total glomeruli in all slides). Recall and F1 scores were somewhat lower, at 80% and 85%, respectively. Visual inspection of the SVM score maps confirms that the vast majority of positive locations contain glomeruli (Fig. 3a, green boxes). Connected areas of the WSI with strong textural similarity to the glomerulus, specifically parts of the kidney medulla, are removed by a simple size-filtering and intensity-thresholding approach, ensuring that false positives do not overwhelm the true positives (Fig. 3b).

Notably, this detection pipeline is robust even to the presence of certain pathological changes within the tissue, as shown by the reasonably good precision achieved even when the pipeline is trained on glomeruli from control mice and applied to glomeruli from STZ-treated mice—85% precision for STZ (*n* = 3 WSIs), versus 93% precision when the same trained classifier is applied to glomeruli from control mice (*n* = 7 WSIs), with standard deviations of 0.0188 and 0.072 respectively (Fig. 3c). Observed recall for glomerulus detection, in fact, showed no significant difference between control and STZ mice, with values of 78% and 82% respectively and standard deviations of 0.106 and 0.0461.

### mrcLBP performance in rat, with multiple stains

**Figure 4:**
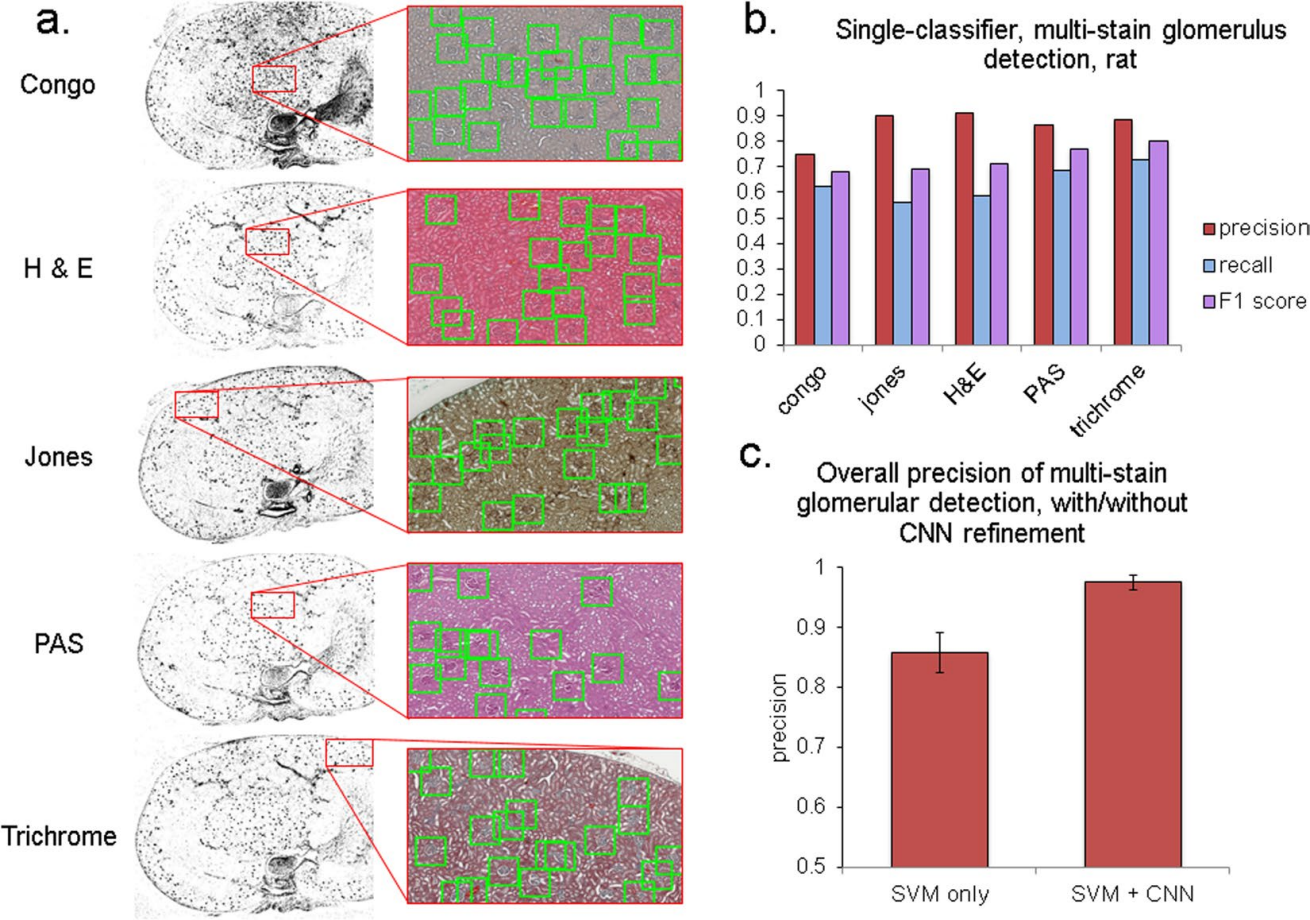
Performance of the glomerular detection pipeline for rat WSIs, with five stains. (**a**) Images showing glom detection results for 5 different stains. From top to bottom: congo, jones, H&E, PAS, and Gömorri trichrome. In this case, an SVM classifier was trained simultaneously on equal numbers of examples from all five stains. Glomeruli are visible as black spots in the score maps (left column) and with overlaid boxes over the original color image (right column). (**b**–**c**) Performance of the mrcLBP method when trained on multiple stain types simultaneously, with or without purification by a deep neural network. Top (**b**), performance of the method after SVM classifier alone in 10 rat WSI. To increase recall, score maps were thresholded lower, admitting more total positives. Bottom (**c**), result of purifying the collected glomerular images with a GoogLeNet neural network model trained on 7099 rat glomeruli in five stains. Precision now exceeds 97.5% for all stains, with minimal decrease in recall (not shown).

Representative results of mrcLBP-based glomerular detection in rat kidney are presented in Fig. 4a, for a classifier trained on an equal mixture of glom(+) and glom(−) images from five separate stains. As can be seen in the zoomed regions, the resulting pipeline is robust to variations in stain type, with an abundance of glomeruli detected in all five cases. Figure 4b shows a summary of results for precision, recall, and F1 score for each of the five types of stained test images (2 WSI each). Total ground truth values for each stain type are 958 glomeruli for congo; 989 for jones; 927 for H&E; 881 for PAS, and 909 for Gömorri trichrome, yielding a total of 4664 glomeruli, with 3450 total positives.

Congo stain showed poorer precision than the other types, possibly due to the much lower visual contrast of the glomeruli in these images (detection is difficult even by eye), though the recall was similar. Jones and H&E slides both showed the highest precision, above 90%, while trichrome-stained slides presented slightly lower precision (88.7%) but offered the highest recall, with 73% of the ground truth gloms detected. Much as the lower precision values for Congo stain are likely due to the very low visual contrast it provides in the glomeruli; the higher recall with trichrome stain is likely a function of the very striking color differentiation it achieves, with glomeruli typically greenish against the red background of tubules and blood vessels.

### Precision improvement of harvested rat glomeruli using neural network classifier

Although neural network approaches can be extremely versatile and achieve very high precision in certain tasks, our experience showed that CNN’s (at least readily available multi-purpose ones such as AlexNet or GoogLeNet, without any special architectural modification for glomerular detection) could achieve classification accuracies of up to 99.8% when trained on thousands of rat or mouse glomeruli. Though impressive, this performance level is still not adequate for glomerular detection by sliding-window analysis of an entire WSI, which may visit tens of thousands of window positions and could therefore still lead to hundreds of false positives. Conversely, many applications would require a precision of much greater than the 90–93% precision values we obtained using mrcLBP with SVM alone. We therefore combined the approaches in tandem, using the mrcLBP feature and SVM first to gather a high-quality set of candidate glomeruli, followed by a trained GoogLeNet model to “purify” the set further.

The results of this tandem purification are summed up in Fig. 4c. Notably, while the recall and F1 values were negligibly decreased by adding the additional classifier, the overall precision reached 97.5% (2929 true positives, versus only 74 false positives), or a 6-fold decrease in the proportion of false positives. The lowest-precision stain was now Jones (95.3%), surpassed by congo (96.6%), trichrome (98.2%), H&E (98.4%) and, highest of all, PAS (99.1% precision). Running the neural network classifier with an Nvidia GeForce 1080 GPU, purification of the glomeruli from each stain was typically achievable in under a minute per WSI.

### mrcLBP: application to human glomeruli

**Figure 5:**
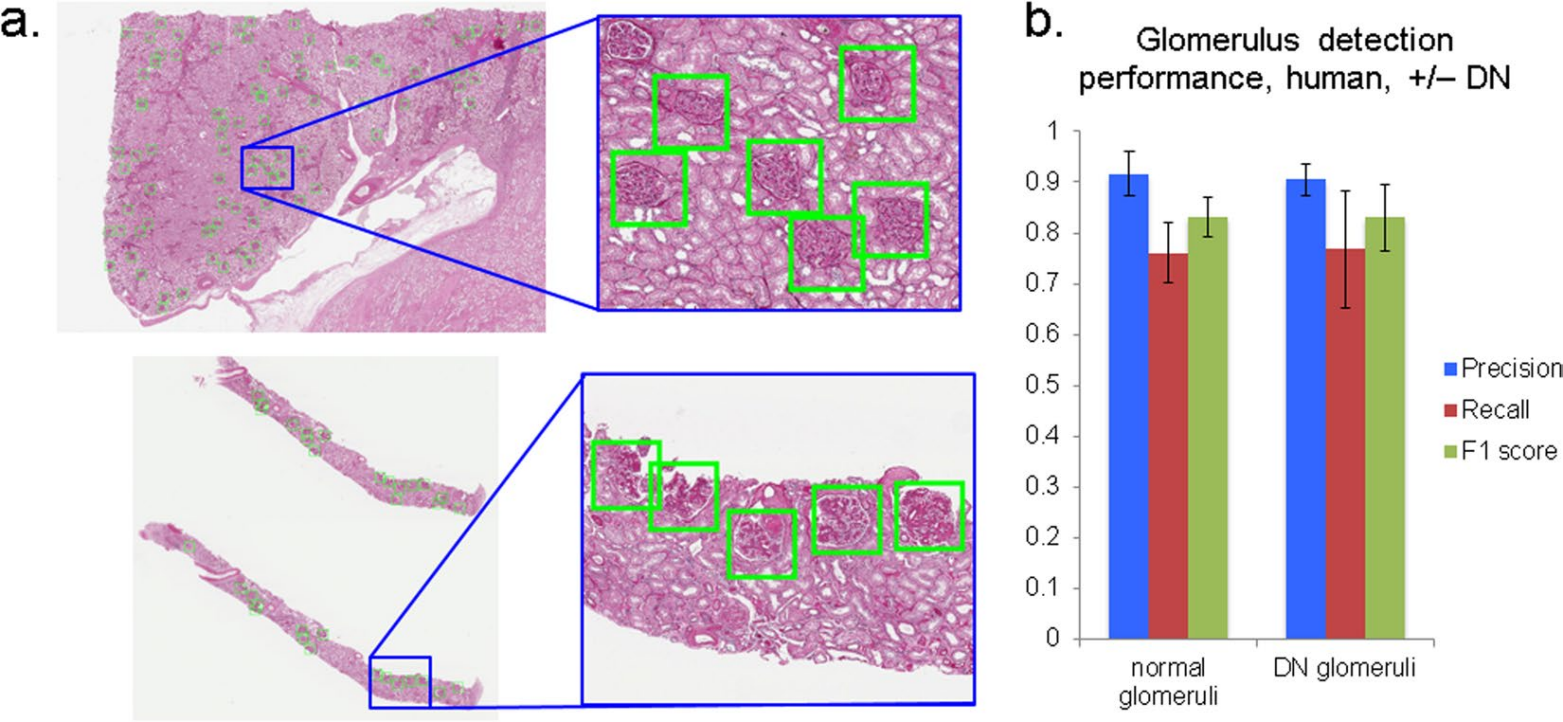
Examples and results from glomerular detection in human WSIs using mrcLBP. (**a**) Examples of successful application of mrcLBP to human renal WSIs, with detected glomeruli (green boxes). Top, renal section. Bottom, needle biopsy. PAS stain. (**b**) Results of detection of glomeruli in human tissue samples. Left group, non-DN patients, from 9 slides containing 1088 glomeruli. Right group, result from DN patients, from 5 slides containing 99 glomeruli.

Applicability of a detection method to human disease data is among the most crucial criteria for clinical and research significance. Therefore, having confirmed the viability of mrcLBP for glom detection in mouse, rat and a variety of staining protocols, we sought to examine its performance in needle biopsies and tissue sections from human kidney. Our training data comprised samples from one individual with glomeruli having no histological abnormalities, 7 individuals with DN, and 1 with IgA nephropathy. Examples of glomerulus detection using the resulting SVM model to test WSIs are shown in Fig. 5a, for a needle biopsy (DN) and tissue section (normal kidney).

Figure 5b shows basic statistics for glomerular detection, on slides from 5 patients with DN (*n* = 5 WSIs) and 3 patients with normal glomeruli (*n* = 9 WSIs). As may be expected for a classifier trained on a mixture of DN and normal glomeruli, the results between the two groups are statistically indistinguishable. Specifically, overall precision values are 91.7% and 90.4% for normal and DN, respectively, with standard deviations (by slide) of 0.083 and 0.061; recall values were 76.1% and 76.7%, standard deviations 0.12 and 0.23; and F1 scores, 0.832 and 0.831, standard deviations 0.0787 and 0.131. Therefore, overall performance of the mrcLBP-trained SVM approach in human glomerular detection appears broadly similar to that demonstrated in mouse and rat WSI.

### Population differences between glomeruli from control and STZ-treated mice

We observed that the mrcLBP-trained SVM classifier, even when trained to detect both DN and healthy glomeruli, still does not detect fully sclerosed (“obsolete”) glomeruli in human tissue. Therefore, we wondered whether this selectivity could be of use in classifying glomeruli by disease state. Due to the readier availability of glomerular images from mouse, and the highly controlled conditions attainable with the mouse STZ model of DN, we decided to re-examine the mouse WSI data using an mrcLBP-trained SVM pipeline, but this time with the aim of classifying harvested glomeruli according to their disease state rather than of glomerular detection.

**Figure 6:**
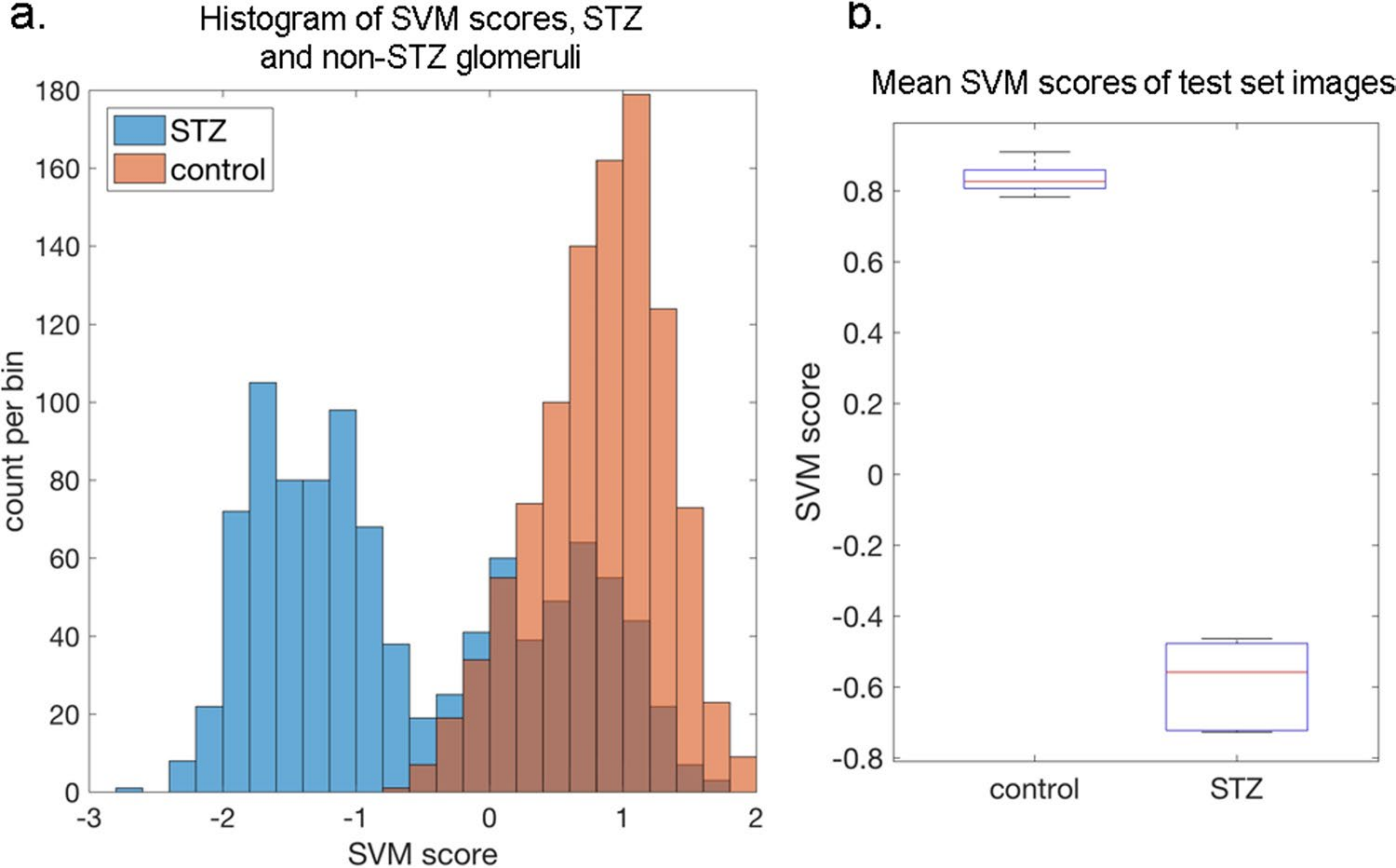
Application of the mrcLBP to a mouse model of diabetic nephropathy reveals significant textural alterations. (**a**) Distribution of aggregated classifier scores after ten random cross-validations. Holdout images from STZ-treated mice (blue bars) show a strongly bimodal distribution of SVM scores, while holdouts from non-STZ-treated mice (orange bars) are unimodal, consistently receiving positive classifier scores. (**b**) Boxplot showing spread of the mean values of STZ and non-STZ scores from all cross-validations.

Using randomized cross-validations of the mouse training data, we examined the distribution of scores produced by an SVM trained to distinguish between glomeruli from control and STZ-treated mice. The aggregated results from 10 such cross-validations (100 holdout images from each class per cross-validation) are presented in Fig. 6a,b. A striking difference in the distribution of the SVM scores for control and STZ-treated mice is immediately apparent in the histogram in Fig. 6a; control glomeruli are almost universally assigned high SVM scores, creating a single large peak to the right of the histogram, while STZ glomeruli are mainly located in a peak at the left of the histogram, corresponding to highly negative SVM scores. Interestingly, however, there is also pronounced bimodality in the STZ distribution, with a substantial minority of STZ glomeruli found in an additional peak that closely overlaps that of the control glomeruli. This finding strongly suggests that the glomeruli in STZ mice belong to at least two distinct sub-populations: one showing pathologic changes and the other remaining (texturally) normal.

Even given the significant bimodality of the STZ scores, the mean values of the STZ and control score distributions show consistent and highly significant separation, as visualized in Fig. 6b. We therefore propose that mrcLBP-trained SVM classifiers are capable of distinguishing, given a sufficient sample size, between control glomeruli and those from STZ mice.

## Discussion

In the foregoing, we have outlined and demonstrated a simple pipeline for the rapid and fully automated extraction of glomeruli from WSI. Our method is easy to implement, requires only a single classification step to achieve performance comparable or superior to the current state-of-the-art, and can process large numbers of WSIs in a short period. Due to its simplicity, a relatively small number of parameters are needed: two intensity thresholds for extraction of renal medulla and for glomeruli, and a size range for filtration of large connected components. We have also shown that the method can be combined with other classifiers, particularly deep neural networks, in order to produce very high precision glomerular detection. Moreover, we have been able to arrive at preliminary results strongly indicating a statistical signature for diabetic nephropathy in a mouse model of the disease.

In contrast, relatively few works so far have directly addressed the problem of glomerular detection and classification simultaneously. Most reported approaches have shown relatively low precision, have required access to extremely large datasets^20,21^, or have made use of biochemical markers. Regarding the latter, in addition to the immunohistochemical and MRI methods earlier alluded to, one particularly novel example involves the use of Fourier-transform IR spectroscopy to assess the progression of diabetic nephropathy in pre-selected glomeruli^40^; however, the sample size was relatively small (68 glomeruli), and the method is likely not conveniently applied to glomerular detection as such.

Other approaches have typically focused on unsupervised morphological processing strategies, for instance edge patching and contour extraction by genetic algorithm^16,19^. In ref.^41^, glomerular diameter measurement was achieved, using a relatively complex unsupervised approach consisting of a combination of median filters, particle size filters, convex hull operations, and aspect ratios. However, only a small number of glomeruli were tested and the process is slow, requiring ~2 minutes to detect and draw a single glomerular boundary. Most such approaches are furthermore dependent on the visual distinctness of the Bowman’s space, something which is readily lost in thicker tissue sections.

Possibly most developed among the glomerular detection methods described to date is the “segmental histogram of gradients” (s-HOG) descriptor in *Kato et al*.^15^, a modification of the standard rectangular histogram of gradients (r-HOG) feature vector. Use of r-HOG for glomerular detection suffers from a very high false positive rate, which s-HOG addresses by creating HOG binning regions individually tailored to glomeruli. The process of constructing the s-HOG feature is however relatively complex, requiring three successive SVM steps, including one to segment the glomeruli and another to eliminate the excessive false positives initially generated by r-HOG. Although speed estimates are not provided by Kato *et al*.^15^, this complexity appears likely to be computationally costly. In contrast, our LBP-based method produces few false positives to begin with, and succeeds using a feature vector of much lower dimensionality (120 vs. 512 for their r-HOG) and only a single SVM step. By way of performance comparison, the reported precision, recall, and F-measure values of the s-HOG method were 87.4%, 89.7%, and 0.866^15^. The precision therefore falls slightly below our method’s typical range of 90–93%, and while our method’s F1 scores and recall values are slightly lower than for s-HOG, we believe that our method presents a valuable alternative in light of its much greater simplicity as well as the special importance of detector precision in the construction of training sets and the gathering of glomeruli for clinical assessment.

More recently, Pedraza *et al*. have announced success with glomerular detection using standard convolutional neural network architectures^42^; yet the training process involved requires up to 33 minutes and the use of a GPU. Also, glomerular detection results from WSIs is not claimed, but rather classification of previously cropped glom(+) and glom(−) images. We note that while it is straightforward to develop high classification performance using such neural networks, the large number of sliding window positions that must be visited to cover an entire WSI combined with the typical sparsity of glomeruli means that classifiers that are excellent for sorting groups of pre-cropped images may nonetheless report many false positives if deployed on WSIs directly.

Indeed, our approach is perhaps notable in that it relegates more complex machine-learning processes such as neural networks to the role of refinement as a way of “purifying” the results obtained by simpler methods. In many cases, the number of false positives is small enough that for some purposes they may be disregarded, or removed manually with good speed. As an additional limitation of the CNN approach, we discovered that neural networks were generally of little use in discerning STZ from control glomeruli, tending to over-train, whereas the arguably far “simpler” feature vector-plus-SVM system was able to find a salient difference in the two groups that is robust to cross-validation and retraining.

A basic distinction among the approaches to automated WSI feature extraction may be usefully described as between “structural”, and “textural” approaches. Texture has long been considered as an important classification feature in medical diagnostics, but mainly with reference to radiotherapy, CT, and MRI scanning^5,43,44^ or, when applied to histopathology, mainly as an aid to cancer grading and the detection of metastases^4,6,45^. Texture features such as LBP have the specific advantages of being simple to extract and “when extracted locally, robust to geometric and illumination changes”, as opposed to structural features which typically require a segmentation step^4^.

One main disadvantage of supervised learning however is the risk of over-training to a particular training set; indeed, development of approaches to recognize tissue structures between numerous histology labs is a major goal^21^. In our case, all training sets were derived from the same institution or lab as the images that were subsequently used for testing, leaving inter-institutional variability as a future subject of study. Based on our success in approaching stain variations with a single classifier, however, as well as the steadily improving standards for WSI acquisition and color normalization, we believe this is a readily attainable aim. Also, although our SVM can be specifically *trained* to detect the difference between STZ and control glomeruli using mrcLBP, the fact that a classifier trained on only control glomeruli (or half of each) detected STZ glomeruli almost as well as control ones suggests that the method does not overlook pathological or otherwise unusual glomeruli; this robustness certainly calls for further testing however.

A number of future extensions of this work suggest themselves. One is glomerular segmentation, since the feature score maps used to detect glomeruli in our approach often already reveal the rough shape of the individual glomeruli. However, increasing the resolution to the point of accurate segmentation of the glomerular boundary presents a significant challenge. Other variations of the LBP feature, such as opponent-color LBP (OCLBP), or Robust LBP (RLPBP)^26^ may assist in this end, for instance by making more efficient use of the color information.

Finally, it is worth noting that in the case of the glomerulus, the strongly distinctive appearance of the glomerular texture and its contrast relative to surrounding tissue offers a major advantage for detection and segmentation, which many other problems in medical imaging and diagnostics will not share. Nevertheless, by demonstrating a path to glomerular detection and classification that can be run even on a home computer, or can aid in extracting large training sets for more intensive statistical analyses, we envision that our results may aid in the development of more general methods of computer-aided-diagnosis in histopathology.

### Availability

Source code and images used to derive the results are available at https://goo.gl/kkpJ1m.
